# Factors Driving the Emigration Intentions of Young Tunisian Medical Professionals

**DOI:** 10.1192/j.eurpsy.2024.1731

**Published:** 2024-08-27

**Authors:** W. Haouari, S. Omri, A. Labyadh, R. Feki, I. Gassara, N. Charfi, J. Ben Thabet, M. B. Maalej, N. Smaoui, L. Zouari, M. Maalej

**Affiliations:** ^1^Psychiatry C, Hedi Chaker university hospital, Sfax, Tunisia

## Abstract

**Introduction:**

Emigration has a substantial impact on Tunisia’s healthcare sector. Graduates, including medical students at different educational levels, as well as general practitioners and specialists, often choose to emigrate. Some do so to pursue further studies abroad, while others seek careers and settlement primarily in Europe. This phenomenon exerts a significant influence on the quality of healthcare systems in their home countries.

**Objectives:**

To evaluate the inclination to emigrate among medical residents employed in Tunisian healthcare institutions and to identify the factors associated with this intention.

**Methods:**

This is a descriptive cross-sectional study conducted among medical residents undergoing their training in various healthcare facilities in Tunisia. The study employed an online questionnaire to assess the degree of satisfaction with various aspects of their professional life and the socio-economic situation in the country, as well as their intention to emigrate. Satisfaction levels were measured using a 4-point Likert scale, ranging from “very dissatisfied” to “very satisfied”.

**Results:**

A total of 50 physicians participated in the survey. Among them, 72% were female, 80% were single, with an average age of 27.72 years at the time of the study. Regarding their professional status, 84% worked in university hospitals, 16% specialized in surgery, 40% specialized in medicine, and 44% were family physicians. The majority were students from the Faculty of Medicine in Sfax (56%), with 30% in Monastir, 8% in Tunis, and 6% in Sousse. The study found that 68% of medical residents expressed an intention to emigrate. Among the participants, 74% were dissatisfied with their working conditions, and 68% were dissatisfied with workplace safety. Additionally, 84% were dissatisfied with their salaries, 40% with their workload, 54% with supervision, and the quality of training provided in their hospitals. The political situation in the country and social security were considered unsatisfactory by 92% and 90% of the participants, respectively. Among the potential reasons studied to explain this emigration phenomenon, working conditions were a factor in 54% of cases, salary in 56%, training in 36%, and quality of life in 56%.

**Conclusions:**

The emigration of young Tunisian medical professionals is driven by a range of factors, including working conditions, salaries, training opportunities, and quality of life. To counteract this phenomenon, it is crucial to enhance these aspects in order to retain these talented individuals in the country and thereby bolster the Tunisian healthcare system.

**Disclosure of Interest:**

None Declared

